# A Novel Nanoprobe for Multimodal Imaging Is Effectively Incorporated into Human Melanoma Metastatic Cell Lines

**DOI:** 10.3390/ijms160921658

**Published:** 2015-09-08

**Authors:** Synnøve Nymark Aasen, Aneta Pospisilova, Tilo Wolf Eichler, Jiri Panek, Martin Hruby, Petr Stepanek, Endy Spriet, Daniel Jirak, Kai Ove Skaftnesmo, Frits Thorsen

**Affiliations:** 1 NorLux Neuro-Oncology Laboratory, Department of Biomedicine, University of Bergen, 5020 Bergen, Norway; E-Mails: synnovenymarkaasen@gmail.com (S.N.A.); kaioveskaftnesmo@gmail.com (K.O.S.); 2 Institute of Macromolecular Chemistry, Academy of Sciences of the Czech Republic, 162 06 Prague, Czech Republic; E-Mails: pospisilova-a@seznam.cz (A.P.); panek@imc.cas.cz (J.P.); mhruby@centrum.cz (M.H.); ppetr.stepanek@gmail.com (P.S.); 3 Department of Clinical Medicine, University of Bergen, 5020 Bergen, Norway; E-Mail: tilo.eichler@k1.uib.no; 4 Molecular Imaging Center, Department of Biomedicine, University of Bergen, 5020 Bergen, Norway; E-Mail: endy.spriet@uib.no; 5 Department of Diagnostic and Interventional Radiology, Institute for Clinical and Experimental Medicine, 140 21 Prague, Czech Republic; E-Mail: daji@medicon.cz; 6 Institute of Biophysics and Informatics, 1st Medicine Faculty, Charles University, 120 00 Prague, Czech Republic; 7 Kristian Gerhard Jebsen Brain Tumour Research Centre, Department of Biomedicine, University of Bergen, 5020 Bergen, Norway

**Keywords:** melanoma brain metastasis, nanoprobe, theranostics, magnetic resonance imaging, fluorescence microscopy, high throughput microscopy, fluorescence lifetime correlation spectroscopy, zeta potential

## Abstract

To facilitate efficient drug delivery to tumor tissue, several nanomaterials have been designed, with combined diagnostic and therapeutic properties. In this work, we carried out fundamental *in vitro* and *in vivo* experiments to assess the labeling efficacy of our novel theranostic nanoprobe, consisting of glycogen conjugated with a red fluorescent probe and gadolinium. Microscopy and resazurin viability assays were used to study cell labeling and cell viability in human metastatic melanoma cell lines. Fluorescence lifetime correlation spectroscopy (FLCS) was done to investigate nanoprobe stability. Magnetic resonance imaging (MRI) was performed to study T_1_ relaxivity *in vitro*, and contrast enhancement in a subcutaneous *in vivo* tumor model. Efficient cell labeling was demonstrated, while cell viability, cell migration, and cell growth was not affected. FLCS showed that the nanoprobe did not degrade in blood plasma. MRI demonstrated that down to 750 cells/μL of labeled cells in agar phantoms could be detected. *In vivo* MRI showed that contrast enhancement in tumors was comparable between Omniscan contrast agent and the nanoprobe. In conclusion, we demonstrate for the first time that a non-toxic glycogen-based nanoprobe may effectively visualize tumor cells and tissue, and, in future experiments, we will investigate its therapeutic potential by conjugating therapeutic compounds to the nanoprobe.

## 1. Introduction

Around 90% of cancer patients die of tumor metastasis [1]. A detailed and accurate diagnosis of cancer metastasis is therefore necessary in the management of metastatic disease [2]. Commonly used imaging techniques in the clinic, such as magnetic resonance imaging (MRI), computerized tomography (CT) and positron emission tomography (PET), play a critical role in diagnosis and therapy of cancer metastasis [2,3,4]. A combined use of imaging modalities (*i.e.*, multimodal imaging) has the potential of combining anatomical and physiological image information, thereby improving our understanding of tumor development and therapeutic responses [5,6].

Recently, increased attention has been given to the establishment of functional nano-scaled materials for the application of combined cancer therapy and diagnostics, also named “nano-theranostics” [7,8,9]. By using such systems, multimodal imaging is able to confirm delivery of therapeutic substances to the tumors and provides a superior visualization of treatment efficacy by real-time monitoring of treatment response [10,11]. An ideal nanoscale drug delivery platform should be biodegradable and non-toxic. The drug load should also occur locally within tumor tissue and reach therapeutic concentrations for a sufficient period of time [12]. Recent developments have advanced the use of theranostic nanomaterials on neoplasms also within the central nervous system (CNS), which may improve treatment of brain metastatic disease in future [13,14]. Polysaccharide-based nanomaterials have drawn notable attention, as they are biocompatible *in vivo* and have the potential to traverse physiological obstacles [15,16,17,18]. Further, optimization of size and surface coating of the nanomaterial may extend the circulation time after intravenous administration compared to standard delivery methods of chemotherapeutic drugs [19]. Moreover, solid tumors spontaneously accumulate biocompatible polymers, polymer micelles, liposomes and nanoparticles less than 200 nm in diameter due to the leaky nature of the newly formed tumor neovasculature. This enhanced permeability and retention (EPR) effect is relatively universal for many solid tumors and allows concentrating nanoparticles to more than one order of magnitude compared to surrounding tissue [20,21].

We have recently developed a nanoprobe for multimodal imaging, composed of glycogen conjugated with gadolinium (Gd-DOTA) and the red fluorescent marker Dyomics-615-NHS (Dy-615) [22]. d-Glucose is normally stored as glycogen in the human body (for instance in muscle and liver tissue), and the use of glycogen as the backbone of a nanoprobe offers several advantages. It is biodegradable and non-toxic to human cells. Furthermore, the abundance, low cost, and wide range of modification possibilities makes glycogen attractive for use in an imaging nanoprobe.

We report here for the first time the application of a glycogen nanoprobe, used to image tumor cells. We demonstrate that the nanoprobe effectively labeled human metastatic melanoma cells *in vitro*, and cellular properties such as viability and migration was not affected after labeling. T_1_ weighted *in vivo* MRI scans showed that the contrast enhancement in subcutaneous tumors obtained by the nanoprobe was comparable to using a contrast agent commonly used in the clinic. Our data suggest that the nanoprobe may likely accumulate in solid tumor tissue due to the EPR effect. The nanoprobe may easily be expanded to a nano-theranostic entity, by conjugating it with a therapeutic substance. The main aim of this study was, however, to show proof-of-principle that the nanoprobe is an effective contrast agent for multimodal imaging, while future experiments will address its theranostic utility, where therapeutic agents will be conjugated to the nanoprobe, and the *in vivo* effects will be studied in our mouse models of metastatic melanoma.

## 2. Results and Discussion

### 2.1. The Glycogen Nanoprobe Is Efficiently Internalized into the Metastatic Melanoma Cell Lines

**Figure 1:**
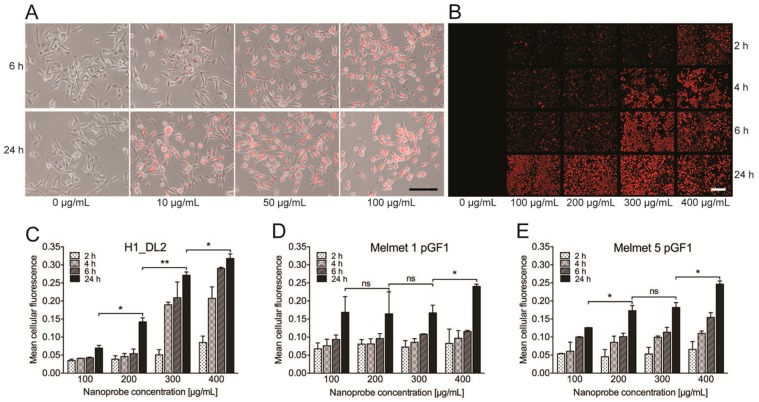
Cellular uptake of the glycogen nanoprobe. (**A**) Fluorescence micrographs overlaid light microscopy images, showing the H1_DL2 cells after being labeled with the glycogen nanoprobe for 6 or 24 h. Scale bar, 100 μm; (**B**) Representative fluorescence micrographs, showing the H1_DL2 cells after being labeled with the glycogen nanoprobe for 2, 4, 6 or 24 h. Similar high throughput experiments were performed for all three cell lines. Scale bar: 100 μm; (**C**–**E**) Quantification of mean fluorescence intensity in the images acquired of the three cell lines by high throughput microscopy. ns: not significant; * *p* < 0.05; ** *p* < 0.01: (**C**) H1_DL2 cell line; (**D**) Melmet 1 pGF1 cell line; and (**E**) Melmet 5 pGF1 cell line.

We first evaluated the uptake of the glycogen nanoprobe into H1_DL2 human melanoma metastatic cells and two normal human fibroblast cell lines (SV-80 and NSF3) by intracellular fluorescence intensity from Dy-615 after labeling the cells with nanoprobe doses ranging from 10 to 100 μg/mL (Figure 1A). After 6 h, H1_DL2 cells incubated with 10 μg/mL nanoprobe had internalized a minor amount of the nanoprobe. Increased concentration of labeling solution resulted in increased uptake of nanoprobe, as seen by elevated fluorescence intensity. Further, incubation for 24 h with the same concentrations showed stronger uptake of the nanoprobe (Figure 1A). We could not detect any uptake of nanoprobe into the two fibroblast cell lines, even at a labeling concentration of 100 μg/mL (Figure S1).

A detailed inspection of the fluorescence images revealed that all the cells were labeled already when using 10 μg/mL of the nanoprobe. However, a rather weak fluorescence was observed for all labeling concentrations, except 100 μg/mL, indicating that higher concentrations should also be evaluated.

Therefore, we increased the labeling concentrations of nanoprobe to between 100–400 μg/mL. Micrographs obtained from live-cell high-throughput imaging indicated that during an incubation time of 24 h all of the cells were effectively and strongly labeled at these concentrations (Figure 1B). The fluorescence intensities from the micrographs were then quantified (Figure 1C–E). In general, there was a dose-dependent as well as a time-dependent increase in mean fluorescence intensities for all cell lines. For the H1_DL2 cells (Figure 1C) and the Melmet 5 pGFI cells (Figure 1E), a statistically significant increase in fluorescence was seen after increasing the labeling concentration to 200 μg/mL. For the Melmet 1 pGF1 cells (Figure 1D), there was no increase in labeling efficacy in the range of 100 to 300 μg/mL (24 h labeling time). Based on these results, we continued to investigate cell viability using a nanoprobe labeling concentration of 200 μg/mL.

### 2.2. The Glycogen Nanoprobe Does Not Affect Short-Term Cell Viability in Vitro

**Figure 2:**
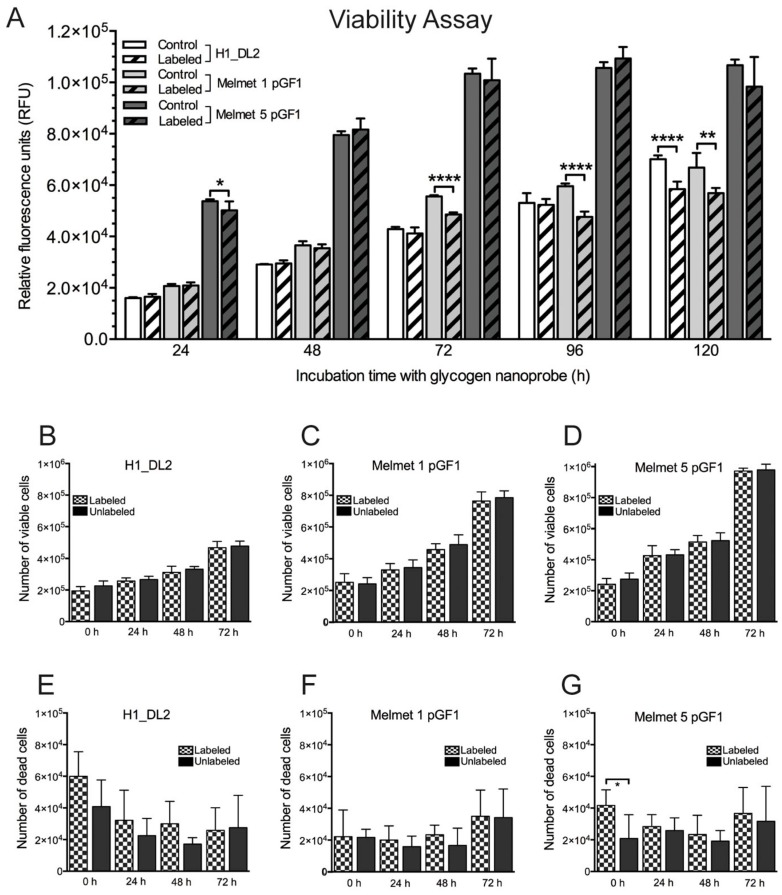
Cell viability in monolayer cultures. (**A**) Monolayer resazurin assay to determine viability of labeled and unlabeled melanoma metastatic cell lines for 120 h. The labeled cells were exposed to 200 μg/mL of the glycogen nanoprobe during the whole time period (for all experiments, *n* = 8; mean ± SD). The relative fluorescence units (vertical axis) were proportional to the number of viable cells. * *p* < 0.05, ** *p* < 0.01, **** *p* < 0.0001; (**B**–**D**) Cell counting assay to determine the number of viable cells in monolayer cultures of labeled and unlabeled cell lines during 72 h. The labeled cells were exposed to 200 μg/mL of the glycogen nanoprobe during the whole time period (for all experiments, *n* = 6; mean ± SD): (**B**) H1_DL2 cells; (**C**) Melmet 1 pGF1 cells; and (**D**) Melmet 5 pGF1 cells; (**E**–**G**) Cell counting assay to determine the number of dead cells in monolayer cultures of labeled and unlabeled cell lines during 72 h. The labeled cells were exposed to 200 μg/mL of the glycogen nanoprobe during the whole time period (for all experiments, *n* = 6; mean ± SD): (**E**) H1_DL2 cells; (**F**) Melmet 1 pGF1 cells; and (**G**) Melmet 5 pGF1 cells.

The survival of cells labeled with 200 μg/mL glycogen nanoprobe was studied using a resazurin viability assay (Figure 2A). From 72 h onwards, Melmet 1 pGF1 cells showed reduced proliferative capacity compared to the unlabeled cell population. This was also seen for Melmet 5 pGF1 cells labeled with 200 μg/mL after 24 h. However, subsequent measurements showed no significant differences at later time points. H1_DL2 cells did not show decreased viability until 120 h of exposure to the glycogen nanoprobe (Figure 2A).

Viability was also assessed by counting live and dead cells, after incubation with 200 μg/mL glycogen nanoprobe for up to 72 h. We found no significant differences in viability between labeled and unlabeled cells for any of the cell lines (Figure 2B–G). Thus, based on the internalization and viability studies, a labeling time of 24 h using a concentration of 200 μg/mL nanoprobe was chosen for subsequent experiments.

### 2.3. The Glycogen Nanoprobe Is Localized in Cytoplasm and Lysosomes

**Figure 3:**
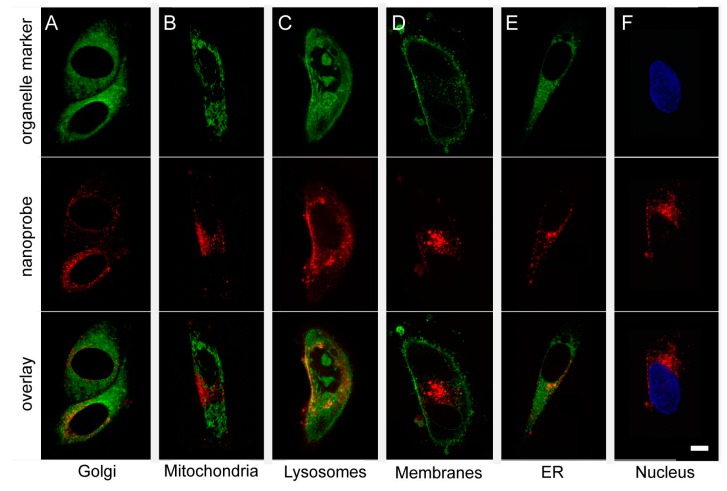
Internalization of the glycogen nanoprobe into organelles within the H1 cells. All organelle probes were conjugated to a green fluorescent dye (**top row**, **A**–**E**) or Hoechst (**F**), while the red fluorescent probe Dyomics-615 was incorporated into the nanoprobe (**middle row**, **A**–**F**). In order to determine co-localization of the nanoprobe with the organelles and thus showing internalization, the red fluorescent images were overlaid the green fluorescent images (**bottom row**, **A**–**E**) or the Hoechst (blue) images (**bottom row**, **F**). (**A**) Staining of Golgi apparatus using the CytoPainter Golgi Staining Kit (Abcam); (**B**) staining of mitochondria using MitoTracker Green (Invitrogen); and (**C**) staining of lysosomes using CytoPainter LysoGreen (Abcam). Areas of co-localization (yellow areas in the overlay picture) could be found; (**D**) Staining of membranes using Wheat Germ Agglutinin Alexa Fluor Conjugate (Thermo Fisher Scientific); (**E**) staining of endoplasmic reticulum using the CytoPainter ER Staining Kit (Abcam); and (**F**) staining of nuclei using Hoechst 33342 (Life Technologies). Scale bar, 10 μm.

To investigate the subcellular localization of the nanoprobe, H1 human metastatic melanoma cells were labeled with the nanoprobe and incubated with markers for different cellular organelles (Figure 3, Table 1). In general, the nanoprobe was localized within the cytoplasm of the cells. Our analysis showed that the only organelle able to internalize the nanoprobe were the lysosomes (Figure 3C, *R* = 0.5614). All other organelles investigated had a random co-distribution with the nanoprobe, and no co-localization could be detected (*R* < 0.5, Table 1).

**Table 1:** Co-localization of nanoprobe to organelles.

Organelle	Mean Pearson’s Correlation Coefficient	*n*	SD
Golgi apparatus	0.0283	2	0.0063
Mitochondria	0.0574	1	0
Lysosome	0.5614	5	0.0849
Plasma membrane	0.0166	5	0.1003
Endoplasmatic reticulum	0.2839	3	0.0535
Nucleus	0.1117	11	0.2049

### 2.4. Intracellular Glycogen Nanoprobe Can Be Detected up to 96 h after Labeling

**Figure 4:**
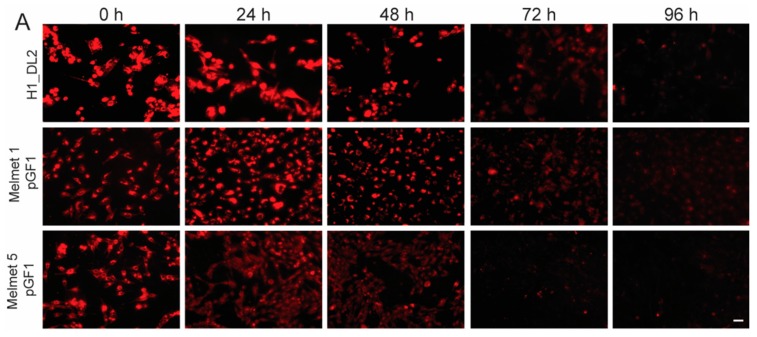

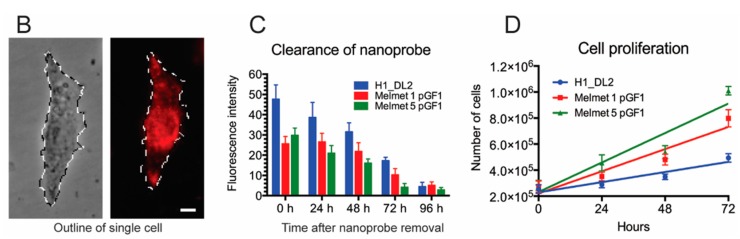
Clearance of the glycogen nanoprobe from cells after removing the labeling solution. (**A**) Representative micrographs demonstrating a gradual decrease in cellular fluorescence intensity for H1_DL2 (**top row**), Melmet 1 pGF1 (**middle row**) and Melmet 5 pGF1 (**bottom row**) cells during four days in culture. Scale bar: 50 μm; (**B**) For each time point, the cellular area of 64 cells (from each cell line) was outlined in Photoshop, as shown in this example. Scale bar: 10 μm; (**C**) The mean fluorescence intensity for each cell was determined, and the mean values of all 64 cells were calculated and plotted for each time point (mean ± SD); (**D**) Cell proliferation for each cell line was determined over a time period of 72 h (mean ± SD). The curves were obtained by linear regression (Graphpad Prism v6).

Nanoprobe clearance was assessed by measuring the decrease in cellular fluorescence intensity over 96 h, after an incubation period of 24 h with 200 μg/mL nanoprobe (Figure 4). In general, the cellular fluorescence intensity declined with increased time (Figure 4A,C). Cellular fluorescence within the cells was measured after outlining the cells, as shown in Figure 4B. In total, 64 cells were measured at each time point for each cell line, and the mean intensity value was calculated. This analysis verified a gradual decrease in fluorescence intensity for all cell lines over a time period of 96 h (Figure 4C). However, around 9% of the nanoprobe was still internalized at that time point. Our study showed an inverse relationship between cell proliferation (Figure 4D) and clearance (Figure 4C), indicating an elevated dilution of nanoprobe in more rapidly proliferating cell lines.

### 2.5. Labeling with the Glycogen Nanoprobe Does Not Influence in Vitro Cell Migration

**Figure 5:**
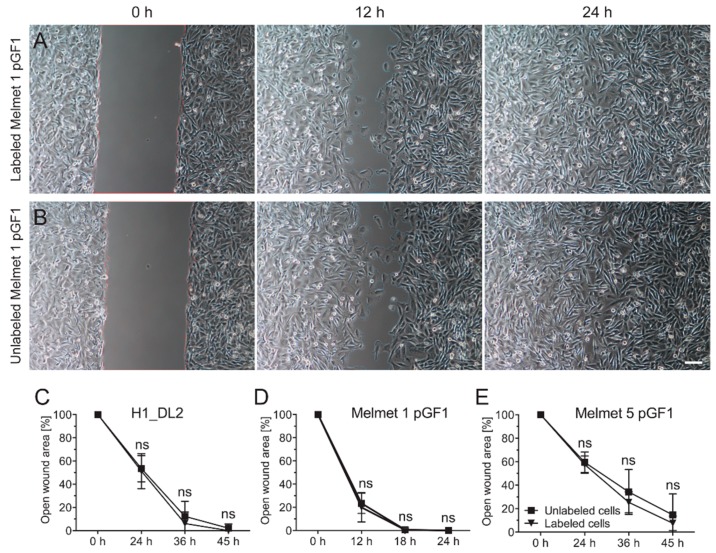
*In vitro* wound healing assay to determine migration capacity. (**A**) Melmet 1 pGF1 cells labeled with 200 μg/mL of nanoprobe; (**B**) unlabeled Melmet 1 pGF1 cells; (**C**) quantification of open wound area of unlabeled and labeled H1_DL2 cells for 45 h; (**D**) quantification of open wound area of unlabeled and labeled Melmet 1 pGF1 cells for 24 h; and (**E**) quantification of open wound area of unlabeled and labeled Melmet 5 pGF1 cells for 45 h. Scale bar: 100 μm; (**C**–**E**) *n* = 8, mean ± SD. ns: not significant.

To study whether labeling with the nanoprobe affected cell migration, a standardized monolayer wound healing assay was carried out (Figure 5). Labeled and unlabeled Melmet 1 pGF1 cells demonstrated similar wound healing capacities, and cell labeling did not influence migration (Figure 5A,B). The wound was closed after 18 h, both for labeled and unlabeled cells (Figure 5D). Similar results were also found for the H1_DL2 and Melmet 5 pGF1 cell lines (Figure 5C,E), as there were no differences in migration between labeled and unlabeled cells. The wounds were closed after 45 h for the H1_DL2 cells, while 15% of the initial area was still open after 45 h for the Melmet 5 pGF1 cell line.

### 2.6. The Surface Charge of the Nanoprobe Is Minimally Affected by pH

The subcellular uptake of the glycogen nanoprobe (Figure 3) could in theory be affected by changes in its surface charge, due to differences in pH within the different cytosolic compartments. Therefore, we measured the zeta-potential of the nanoprobe as a function of pH. We found that the zeta-potential of the glycogen nanoprobe was slightly positive and decreased with increasing pH (obtained values of zeta-potential were 16.7 ± 0.85, 13.8 ± 0.72, 7.28 ± 0.47 and 5.10 ± 0.74 mV, for pH 5.03, 6.00, 6.99 and 7.39, respectively). This can be explained by presence of unreacted amino groups.

### 2.7. The Nanoprobe Is Stable in Blood Plasma for at Least 60 min

We measured nanoprobe stability over time by fluorescence lifetime correlation spectroscopy (FLCS). In general, our results consistently indicated that the glycogen nanoparticles were stable for at least 60 min in human blood plasma.

We first measured the labeled glycogen in physiological saline, and obtained a recovered diffusion constant of about 4 μm^2^/s, which is around two orders of magnitude lower than what a free dye of the size of Dy-615 would have in water. A closer inspection of the FLCS curve revealed that there was a small fraction of faster diffusing particles, with a diffusion coefficient D around 30–40 μm^2^/s, likely due to a fraction of unbound Dy-615 diffusing in the solution. That means that a small fraction of Dy-615 was non-covalently linked to the glycogen particles and preserved partly its mobility.

We then measured pure human blood plasma, and showed that the autofluorescence intensity after 640 nm laser excitation was very small, less than 5% of the intensity of labeled glycogen under the same conditions, and could therefore be neglected in the subsequent measurements of plasma–glycogen mixtures. The average diffusion coefficient was around 60 μm^2^/s.

The labeled glycogen and blood plasma were then mixed directly in a one-chamber microscope dish, and FLCS was performed. The observed diffusion clearly consisted of two major components. One component was practically identical to the major component of the labeled glycogen in physiological saline (D around 4 μm^2^/s). The other component was faster with a diffusion constant similar to plasma (D around 60 μm^2^/s), and was likely due to a non-covalently linking of a small Dy-615 fraction released from the labeled glycogen to plasma proteins (as seen in saline).

Most importantly, the diffusion of the glycogen nanoparticles themselves (fraction with diffusion constant of about 4 μm^2^/s) did not show any change during 60 min after mixing.

### 2.8. The Glycogen Nanoprobe Is an Effective Contrast Agent in Vitro

**Figure 6:**
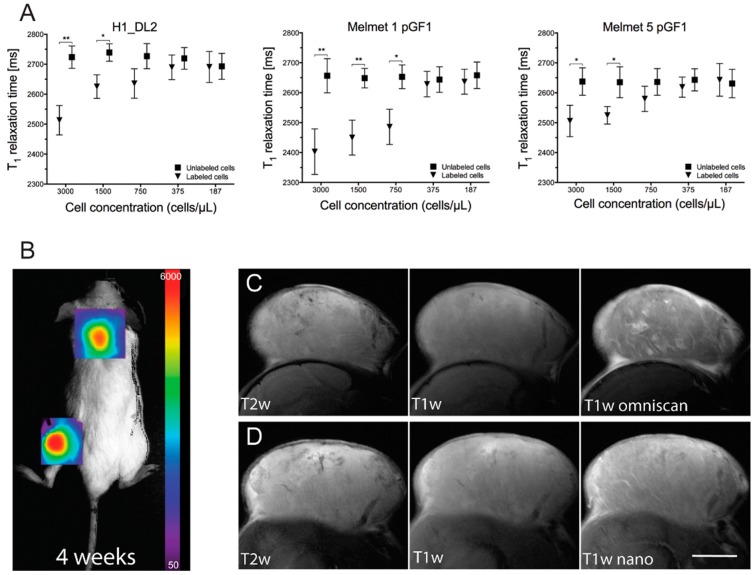
*In vitro* relaxometry and *in vivo* tumor imaging. (**A**) T_1_ relaxation times for phantoms containing labeled and unlabeled H1_DL2 cells (**left**), labeled and unlabeled Melmet 1 pGF1 cells (**middle**), and labeled and unlabeled Melmet 5 pGF1 cells (**right**). The different cell concentrations used are shown on the horizontal axis. Mean ± SD, *n* = 3. * *p* < 0.05; ** *p* < 0.01; ms: milliseconds; (**B**) Representative bioluminescence image of tumor growth in one of the NOD/SCID mice four weeks after injection of H1_DL2 cells in the neck and flank area; (**C**) Representative axial MR images of the neck tumors in the same mouse as shown in (**B**), eight weeks after tumor cell injection. T_2_ weighted image (**left**), T_1_ weighted image before contrast injection (**middle**), and T_1_ weighted image after injecting Omniscan (**right**); (**D**) Magnetic resonance (MR) images of the same mouse as in (**C**), using the nanoprobe as contrast agent. T_2_ weighted image (**left**), T_1_ weighted image before contrast injection (**middle**), and T_1_ weighted image after injecting nanoprobe (**right**). Scale bar, 5 mm.

To study whether cell labeling with the glycogen nanoprobe reduced the T_1_ relaxation times, we conducted an *in vitro* relaxometry experiment with MRI phantoms (Figure 6A). For Melmet 1 pGF1 cells, we detected a statistically significant reduction in T_1_ relaxation times for labeled cells (with 200 μg/mL nanoprobe) down to 750 cells/μL, as compared to the phantoms with unlabeled cells. For phantoms with H1_DL2 and Melmet 5 pGF1 cells, labeled cells reduced the T_1_ relaxation down to cell concentrations of 1500 cells/μL, as compared to the control phantoms (Figure 6A). The data also suggested that a concentration of 750 cells/μL of labeled H1_DL2 and Melmet 5 pGFI cells could reduce the relaxation times, however this was not statistically significant.

### 2.9. The Glycogen Nanoprobe Effectively Enhances MRI T_1_ Contrast in Subcutaneous Tumors

Tumor take and development was verified by bioluminescence imaging (BLI) four weeks after subcutaneous injection of H1_DL2 cells into the mice, and tumor necrosis was not observed at that time point (Figure 6B). T_2_ weighted MRI images acquired eight weeks after tumor cell injections showed tumor edema and necrotic areas within the tumors (Figure 6C-left, Figure 6D-left). Administration of Omniscan showed enhanced tumor contrast on T_1_ weighted images (Figure 6D-right), most likely due to leaky tumor blood vessels, compared to T_1_ weighted images obtained before contrast injections (Figure 6D-middle). In addition, contrast enhancement of the tumors on T_1_ weighted scans was clearly detected after administration of the nanoprobe (Figure 6D-middle, right).

We then used the T_1_ weighted images to calculate the contrast noise ratio (CNR) in tumor *versus* normal tissue adjacent to the tumor on two mice scanned with both contrast agents. We found that the mean CNR value was 42.8 after administration of Omniscan, and 49.5 after administration of the glycogen nanoprobe. This indicated that the contrast efficiency was comparable between Omniscan and the nanoprobe.

### 2.10. Discussion

In the current study, we show that our novel theranostic, glycogen based nanoprobe effectively and safely labeled several different human melanoma metastatic cell lines. Nanoprobe labeling did not influence the cellular behavior, labeled cells could easily be visualized *in vitro* with microscopy and MRI, and used as an effective *in vivo* MRI contrast agent. To our knowledge, the use of glycogen in a multimodal imaging nanoprobe has not been reported previously in tumor cell imaging.

The fluorescence microscopy experiments showed that our nanoprobe was efficiently internalized into three different human melanoma metastatic cell lines, and the cellular uptake increased with elevated incubation times and doses (Figure 1). The amount of cellular internalization is commonly dependent on cell type, labeling time, and also on several properties of nanomaterials such as size, surface characteristics, morphology and charge [23,24,25]. Cellular uptake is reported to be dependent on the size of the nanoparticles, and materials around 50 nm (our nanoprobe is 54.4 nm in diameter) are efficiently endocytosed by cells [24,26,27]. We observed variations in fluorescence intensity and thus nanoprobe uptake among the three cell lines investigated. Others have also demonstrated varying internalization rates, using a gadolinium-based nanomaterial to label a larynx cancer cell line, a glioblastoma cell line and healthy lymphocytes [28]. The observed variations in cellular uptake were ascribed to differences in cell membrane permeability among the cell lines, which could also be the case in our experiments.

The uptake of nanoprobe by the cells is likely due to a combination of nonspecific adsorptive endocytosis, clathrin-mediated endocytosis, macropinocytosis and dynamin-dependent endocytosis, as previously described for such materials [29]. These uptake mechanisms do not require specific receptors on the cells, and the zeta potential plays eventually an important role in the uptake. Molecules with positive zeta potential (our nanoprobe is weakly positive) would be more effectively internalized into the cells than neutral or negatively charged particles [29].

In general, labeling of our metastatic melanoma cell lines did not affect cell viability, indicating that the nanoprobe itself was not toxic to the cells (Figure 2). Labeled Melmet 5 pGF1 cells exhibited a slightly reduced capacity to reduce the non-fluorescent resazurin to the highly fluorescent resorufin at 24 h. However, no differences were observed at later time-points. Melmet 1 pGF1 cells showed a slightly reduced viability after 72 h of incubation with the nanoprobe by the resazurin assay. It is likely that cells exposed to the nanoprobe may replace intracellular glycogen with the nanoprobe [22]. Our findings may indicate that the glucose cycle in the Melmet 1 pGF1 cell line was not as effective in utilizing the glycogen as well as in the other two cell lines.

Our nanoprobe was mainly localized in the cytoplasm of the cells, which is in agreement with other studies showing localization of native glycogen within mammalian cells [30]. The signal from Dy-615 was concentrated around the nucleus and scattered throughout the cells in focal areas of varying sizes (Figure 3). In confocal images, co-localization between red and green stained particles would be observed as yellow areas. Since we commonly observed weak yellow areas in our images, a more thorough analysis was performed. The analysis of the subcellular localization of the nanoprobe demonstrated accumulation in lysosomes after 24 h. The nanoprobe was only randomly co-localized with the other markers investigated (Table 1). Accumulation of glycogen into lysosomes is in line with previous *in vivo* experiments on rats [31], and lysosomes have been referred to as the most common organelle involved in intracellular degradation of nanomaterials [26,32].

Our study showed a gradual clearance of the nanoprobe during 96 h (Figure 4). After 72 h, a relatively high amount of fluorescent signal could still be detected, indicating that therapeutic compounds attached to the nanoprobe in future experiments also may be internalized and exert its effects within the cells for several hours. Due to limitations in resolution of our confocal instruments, we could not investigate whether Dy-615 was still attached to the probe at this time point, or if the nanoprobe had been degraded inside the cytoplasm.

Our measurements of the zeta potential showed that the nanoprobe itself exerted a relatively stable, very slightly positive charge (in fact nearly electroneutral—within the range ±30 mV) at different pH levels. According to the manufacturers information on Dyomics probes in general, the fluorescence intensities from these probes are not dependent on variations in pH, as the structure of carboxyl group-conjugated Dyomics 615 does not contain acidobasically active groups that would have pK close to physiological pH range 5–7.4 (the amino groups have very low basicity due to aromatic conjugation). Taken together, the observed decrease in fluorescence intensity over 96 h is likely not due to quenching of the fluorescent signal, but due to clearance of the nanoprobe from the cells. The nanoprobe may be entrapped within secretory vesicles in the Golgi apparatus, before being exocytosed by the cells [20], although we did not observe co-localization between Golgi and nanoprobe in our experiments.

In addition to the suggested lysosomal degradation of the nanoprobe, we also showed that there was a negative correlation between the cell proliferation rate (Figure 4D) and temporal decrease in fluorescence intensity (Figure 4C), indicating that the observed decrease in fluorescence was also caused by dilution of the nanoprobe due to cell proliferation.

There were no differences in migration between unlabeled and labeled cells. This indicates that cellular features such as cytoskeletal structure and cell polarization are not affected due to exposure of the nanoprobe [33,34]. This finding is in line with other studies investigating effects of nanomaterials on cancer cell lines [35].

The *in vitro* MRI study showed that we were able to detect 750 labeled cells/μL after labeling with 200 μg/mL nanoprobe. Terreno and colleagues evaluated the number of Gd-HPDO3A labeled rat hepatocarcinoma cells visible by MRI in agar phantoms [36]. They incubated the cells with contrast agent in standard culture conditions for 16 h (5–100 mM), and showed that the minimum number of cells they were able to detect by MRI was 5000 cells/μL. Thus, our nanoprobe was up to 6.7 times more sensitive *in vitro*, at the concentrations we used.

We observed by fluorescence microscopy that a nanoprobe dose of only 10 μg/mL in the growth medium labeled all cells, however the fluorescence intensity was too low to be effective in further *in vitro* experiments. We did not study if other labeling concentrations would improve the detection limit by MRI, although it is likely that higher labeling concentrations could improve the sensitivity of the MRI methods.

*In vivo* MRI showed that the glycogen nanoprobe increased contrast on T_1_ weighted images after administering the nanoprobe intravenously, which was comparable to what was seen after administering Omniscan to the same tumor. This was also confirmed by the CNR analysis. Thus, we demonstrated a proof-of-principle that our nanoprobe can be used as a contrast agent in MRI, in addition to being an excellent probe for fluorescent imaging.

We have previously described that a 200 μm diameter brain metastasis in our animal/tumor model consists of around 350–400 tumor cells [4]. Here, we show that we were able to detect down to 750 cells/μL in the agar phantoms. In our animal/brain metastasis model [34], brain lesions with a diameter of 300 μm would thus contain around 1300 tumor cells, and should likely be detected by T_1_ weighted MRI in experiments *in vivo*, if successfully labeled with our nanoprobe.

We did not investigate by *ex vivo* fluorescence whether the nanoprobe was internalized in the tumor cells *in vivo*, as we did not possess suitable tumor tissue for such analysis, and the main focus in this work was to perform a detailed *in vitro* characterization of the novel nanoprobe. Future studies will address this issue.

Glycogen is relatively inactive to amylase present in the blood, and can thus survive transportation in the bloodstream without being immediately eliminated, as the molecular weight is above the renal threshold [22], which our FLCS study also supports. The FLCS allowed us to track the size of the fluorescently labeled nanoparticles even in a complex environment such as blood plasma. We observed that the slow-moving fraction of the fluorescence (*i.e.*, the intact nanoprobe) did not change its diffusion coefficient within 60 min incubation with blood plasma, implying that the nanoprobe did not change its size due to degradation within this time frame. Our data, however, suggests that when the nanoprobe is internalized in cells, degradation within the lysosomes may occur. Due to the enhanced permeability and retention effects present in most tumors, the nanoprobe may be retained within tumor tissue for several hours after its arrival. Thus, any pharmaceuticals incorporated into the nanoprobe would likely be located within the tumor cells for a corresponding period of time, offering a benefit over conventional chemotherapeutical approaches.

The glycogen nanoprobe would be of particular interest to us in future studies of drug delivery to brain metastasis in our animal brain tumor models [4,35]. The blood brain barrier (BBB) plays an important role in the treatment of brain metastasis, as delivery of chemotherapeutics to small tumors is inefficient due to an intact vascular barrier in the brain. We have previously shown that a wide variety of imaging tracers, with molecular weights ranging between 0.24 and 65.55 kDa, are able to leak across the BBB at various stages of brain metastasis development [2,4]. Even if the size of our nanoprobe is in the order of 10 MDa [22], further modifications of the probe by, for instance, attaching suitable antibodies to the surface, could facilitate receptor-mediated transcytosis of the nanoprobe through the vascular endothelium of the brain [37].

In this study, we show that two different normal human fibroblast cell lines (SV-80 and NSF3) did not accumulate the nanoprobe at labeling concentrations up to 100 μg/mL. This is in line with other studies, which have shown that nanoparticles may accumulate to more than one order of magnitude in tumors compared to surrounding tissue, due to the EPR effect [20,21]. This implies that while in normal tissues the probe is in the vessels (in blood volume) physically separated from the surrounding tissue by the vessel wall, in tumor tissue it is present mainly in interstitial space in close touch with the tumor cells that subsequently may take up the nanoprobe.

Uptake of nanoprobe into different animal organs, and clearance *in vivo* was not evaluated. Others have injected glycogen into rabbits and found that within a few hours, glycogen had diffused from the bloodstream to other organs. Further, it was suggested that glycogen used by this route of administration could hold a clinical value, as it was considered harmless [38]. It has been demonstrated previously by MRI that our glycogen nanoprobe was eliminated through the mouse kidneys. Six hours after intravenous injections, only a negligible amount of the nanoprobe was found within the kidney of the animals, which suggested minimal renal burden [22].

## 3. Experimental Section

### 3.1. Glycogen Nanoprobe

The process of synthesis as well as a detailed characterization of our glycogen nanoprobe has been described previously [22]. Briefly, natural glycogen was modified to an amino group-bearing derivative, and then it was conjugated with 0.33 wt % fluorochrome Dyomics-615-NHS ester (Dy-615) with a fluorescence emission maximum at 643 nm [39] and 3.19 wt % of the MRI T_1_ contrast agent gadolinium (Gd-DOTA). It was demonstrated that the nanoprobe holds a spherical shape with a mean diameter at 54.4 nm, has a weakly positive charge and a uniform distribution of contrast agent.

### 3.2. Cell Lines and Cell Culture

The H1 cell line was developed in our laboratory from a resected brain tumor obtained from a patient with metastatic melanoma [40]. The H1 cells were transduced with two lentiviral vectors, encoding Dendra (a GFP variant) and Luciferase, to obtain the H1_DL2 cell line [35]. The Melmet 1 and Melmet 5 cell lines were established from human metastatic melanomas in subcutaneous tissue and a lymph node, respectively [41]. The Melmet 1 and Melmet 5 cells were transduced with a lentiviral vector, encoding CopGFP (a GFP variant, Systems Biosciences, Inc., Mountain View, CA, USA) and Luciferase, resulting in the Melmet 1 pGF1 and Melmet 5 pGF1 cell lines. Two normal human fibroblast cell lines were also used, the SV-80 lung fibroblast cell line (obtained from CLS Cell Line Services GmbH, Eppelheim, Germany) and the NSF3 skin fibroblast cell line (developed in our lab). We obtained written consent before tumor material was collected. The Regional Ethical Committee (#013.09) and the Norwegian Directorate of Health (#9634) approved the tissue collection and storage.

All cells were grown in Dulbecco’s Modified Eagle Medium (DMEM, Sigma-Aldrich Inc., St. Louis, MO, USA) supplemented with 10% heat-inactivated newborn calf serum (Thermo Fisher Scientific, Waltham, MA, USA), 4 times the prescribed concentration of nonessential amino acids (BioWhittaker, Verviers, Belgium), 2% l-glutamine (BioWhittaker), penicillin (100 IU/mL, BioWhittaker), and streptomycin (100 μL/mL, BioWhittaker), hereafter referred to as complete DMEM. The cells were kept in a standard tissue culture incubator at 37 °C with 100% humidity and 5% CO_2_. The growth medium was exchanged twice a week.

### 3.3. Cellular Internalization of the Nanoprobe

The Melmet 1 pGF1, Melmet 5 pGF1, H1_DL2, SV-80 and NSF3 cell lines were grown in 6-well plates (Nunc, Roskilde, Denmark) at a density of 10^5^ cells/mL in a total volume of 2 mL complete DMEM. After 24 h of incubation, the growth medium was replaced with complete DMEM containing the glycogen nanoprobe at final concentrations of 10, 50 or 100 μg/mL. Dishes with unlabeled cells were used as negative controls. After 6 or 24 h of incubation with the nanoprobe, the cells were fixed using 4% paraformaldehyde (PFA, Thermo Fisher Scientific) diluted in phosphate-buffered saline (PBS, Sigma-Aldrich). Subsequently, the cells were studied by fluorescence microscopy with a Nikon TE2000 inverted microscope (Nikon Instruments Inc., Melville, NY, USA) using a 20× ELWD Plan Fluor objective. Micrographs were acquired using the NIS Elements software (64 bit version, Nikon Instruments Inc.), with identical settings (gain 1.7, exposure time 600 ms) for all fluorescence images. The micrographs were processed using Photoshop CS5 (Adobe Systems Inc., San Jose, CA, USA).

In a second internalization study, the three melanoma cell lines were seeded into the wells of three 96-well plates (Nunc) at a density of 4 × 10^3^ cells/mL in a total volume of 100 μL complete DMEM and incubated for 24 h. The medium was then substituted with growth medium containing the nanoprobe at concentrations of 100, 200, 300 or 400 μg/mL (*n* = 8 wells per concentration), and incubated for 2, 4, 6 or 24 h. Wells with unlabeled cells were used as negative controls. At the different time points, the wells were washed with preheated complete DMEM (37 °C), and imaged with a BD Pathway 855 high content imager (BD Biosciences, San Diego, CA, USA) using the 10× objective. The mean cellular fluorescence within each image was determined using CellProfiler v2.1.1 (Broad Institute, Cambridge, MA, USA).

### 3.4. Cell Viability

We performed a monolayer resazurin cell viability assay to study cell proliferation during incubation with the nanoprobe. The Melmet 1 pGF1, Melmet 5 pGF1 and H1_DL2 cells were each seeded into the wells of a 96-well dish (Nunc) at a density of 5 × 10^3^ cells per well in 100 μL complete DMEM. After 24 h, the growth medium was exchanged with complete DMEM containing the nanoprobe in final concentrations of 100, 200, 300 or 400 μg/mL. Unlabeled cells were used as negative controls. After another 24 h of incubation, Resazurin solution (0.1 mg/mL, Sigma-Aldrich) was added at a volume corresponding to 10% of the total volume within each well. Following incubation for 4 h, the absorbance was measured at dual mode 560/590 nm with a scanning multiwell spectrophotometer (Victor 3 1420 multi-label counter, Perkin Elmer, Waltham, MA, USA), using WorkOut v2.0 software (Dazdaq Solutions Ltd., East Sussex, UK). This procedure was repeated on Days 2, 3, 4 and 5. The obtained measurements were in relative fluorescence units (RFUs) and based on six parallel samples. Monolayer cell viability was also determined by counting live and dead cells after incubating the cells with the nanoprobe. The cell lines were seeded into three six-well plates (Nunc) at a cell density of 1 × 10^5^ cells/mL in 2 mL of complete DMEM. After an incubation period of 24 h, the growth medium was replaced with complete DMEM containing nanoprobe at a concentration of 200 μg/mL. Unlabeled cells were used as negative controls. The numbers of live and dead cells were then counted 24, 48 and 72 h after labeling, by adding 0.5 mL trypsin EDTA (BioWhittaker) and mixing 1:1 with growth medium. The cell numbers were determined using a Countess Automated Cell Counter (Invitrogen, Paisley, UK), according to the manufacturer’s instructions. The experiment was done in triplicate.

### 3.5. Subcellular Internalization of the Nanoprobe

We then studied whether organelles within the tumor cells were able to internalize the nanoprobe. In total, 5 × 10^4^ H1 cells were distributed into five poly-l-lysine coated (Sigma-Aldrich Inc.) 25 mm μ-Dishes (Ibidi GmbH, Munich, Germany) and incubated for 24 h. The growth medium was then exchanged with 1 mL 200 μg/mL glycogen nanoprobe diluted in complete DMEM and incubated for another 24 h. We stained the Golgi apparatus using the following method: A 1× Assay Solution (Abcam ab139483, Cambridge, UK) was prepared, containing 1 mL 10× Assay Buffer 1 (Abcam), 0.2 mL 50× Assay Buffer 2 (Abcam) and 8.8 mL milliQ water. 100 μL of this solution was then mixed with 50 nM Golgi Green Detection Reagent (Abcam), before 100 μL of the solution was preheated (37 °C) and added directly into the dish containing adhered cells. The lysosomes were stained by mixing 20 μL LysoGreen Indicator (Abcam ab112136) with 10 mL Live Cell Staining Buffer (Abcam), heated to 37 °C and 100 μL of the solution was added to the cell sample. The plasma membrane was stained using Wheat Germ Agglutinin (WGA), by dissolving 1 mg of Alexa Fluor^®^ 488 Conjugate (Thermo Fisher Scientific) in 1 mL PBS (Sigma-Aldrich), before diluting this to a working solution of 5 μg/mL. 100 μL of this solution was then preheated (37 °C) and added to the cell sample. We stained the endoplasmatic reticulum (ER) by mixing 1 mL Green Detection Reagent (Abcam ab139481) with 1× Assay Solution (Abcam) and adding 100 μL of this preheated (37 °C) solution to the cell sample. For staining of the nucleus, the cells were incubated at room temperature for 30 min in growth medium containing 1 μg/mL Hoechst 33342 (Life Technologies). All cells were then washed twice with PBS and fixed with 2 mL 4% PFA (Thermo Scientific).

Mitochondria staining was carried out by incubating the cells with 150 nM MitoTracker^®^ Green FM (Invitrogen) for 30 min before the labeling solution was replaced with complete DMEM. Live cell imaging was performed using a Leica TCS SP5 confocal microscope (Leica Microsystems, Wetzlar, Germany), with excitation wavelengths of 408, 488 and 633 nm, and a 63× HCX PL Apo objective with NA 1.4. All images were processed using Imaris V 7.6.3 (Bitplane AG, Zürich, Switzerland). Co-localization was investigated in 3D image stacks with the co-localization plugin after the cells were cropped to optimal size and the auto-threshold function was applied. The Pearson’s correlation coefficient was measured and the average of all values for each organelle marker was determined. The Pearson’s correlation coefficient, R, describes the relationship in intensity distribution between two color channels, and the value ranges from −1 to 1. Values between −1 and 0.5 suggest no co-localization, *R* > 0.5 suggest some degree of co-localization and *R* = 1 suggests complete co-localization [42].

### 3.6. Nanoprobe Clearance

The Melmet 1 pGF1, Melmet 5 pGF1 and H1_DL2 cells were seeded into six-well plates at a density of 1 × 10^5^ cells/mL in 2 mL complete DMEM, and incubated for 24 h. The growth medium was then replaced with complete DMEM containing nanoprobe at a concentration of 200 μg/mL. After a further incubation period of 24 h, the labeling solution was exchanged with complete DMEM without nanoprobe. Four micrographs of the labeled cells were captured every 24 h for four days using a Nikon TE2000 inverted microscope (Nikon Instruments Inc., NY, USA) with a 20× objective. In each micrograph, 16 single cells were processed to find the average cellular fluorescence intensity using Photoshop CS5 (Adobe Systems Inc., San Jose, CA, USA).

### 3.7. Study of in Vitro Cell Migration

To study the effects of the nanoprobe on cell migration, a monolayer wound healing assay was performed. A silicone culture insert (Ibidi GmbH, Martinsried, Germany) was positioned into each well of a 4-well μ-Slide (Ibidi GmbH). 70 μL of H1_DL2, Melmet 1 pGF1 and Melmet 5 pGF1 cell solution at a concentration of 5 × 10^5^ cells/mL was then added into each half of the culture inserts. The cells were allowed to grow to confluence before the culture insert was removed, resulting in a cell free gap with a width of 500 μm within the culture. All chambers were washed twice with preheated (37 °C) complete DMEM before the chambers were filled with 500 μL growth medium containing 200 μg/mL nanoprobe. As negative control, chambers were filled with 500 μL growth medium without the nanoprobe. Immediately afterwards, cell migration into the wound was studied by time lapse microscopy for 60 h using a Nikon TE2000 inverted microscope (Nikon Instruments Inc., Melville, NY, USA) equipped with an incubator holding 37 °C, 100% humidity and 5% CO_2_. Images were then processed and analyzed using ImageJ v1.46a freeware (National Institutes of Health, Bethesda, MA, USA).

### 3.8. Measurement of Zeta-Potential

The glycogen nanoprobe was diluted in 0.01 M phosphate buffer to obtain 0.1% (wt) solution. The pH of each sample was adjusted to accurate value using few microliters of diluted HCl or NaOH solution. Measurement was performed with Zetasizer Nano-ZS instrument (Malvern Instruments Ltd., Malvern, UK) that computes the zeta potential through the Henry equation.

### 3.9. Measurement of Stability of the Glycogen Nanoprobe in Blood Plasma by Fluorescence Lifetime Correlation Spectroscopy (FLCS)

FLCS is a variant of fluorescence correlation spectroscopy (FCS), which uses differences in rates of fluorescence intensity decays to obtain separate FCS autocorrelation functions (ACFs) of individual fluorophore populations in a mixture [43]. We used this technique to study the stability of the labeled glycogen nanoprobe in human blood plasma. The FLCS experiments were performed using an Olympus IX83 confocal laser scanning microscope with FluoView 1200 software (Olympus Corporation, Tokyo, Japan), extended with a FLIM/FLCS upgrade kit and SymphoTime64 software (PicoQuant GmbH, Berlin, Germany). The measurements were performed with a 640 nm pulsed laser with repetition frequency of 40 MHz and with PMA hybrid detectors.

First, a reference measurement of the labeled glycogen nanoprobe with a concentration of 500 μg/mL in physiological saline was performed; Second, a measurement of human blood plasma (obtained from Sigma-Aldrich Ltd., Prague, Czech Republic) was performed with the same setup as for the reference experiment. The labeled glycogen nanoprobe and blood plasma were then mixed directly in a one-chamber microscope dish (with the same final nanoprobe concentration, *i.e.*, 125 μg/mL). Several measurements were performed, one immediately after mixing, and then 20, 40 and 60 min later. For the measurements in blood plasma, the FLCS curves were fitted with a two component model, to obtain the recovered diffusion constants.

### 3.10. In Vitro MR Relaxometry

The Melmet 1 pGF1, Melmet 5 pGF1 and H1_DL2 cells were prelabeled with nanoprobe (200 μg/mL) in monolayer for 24 h, as described in previous sections. A 2% Difco Agar Noble solution (BD, Franklin Lakes, NJ, USA) was prepared in autoclaved water, microwaved and placed in a preheated waterbath (50 °C). The agar solution was further mixed 1:1 with growth medium containing prelabeled cells. The agar/cell solution was then aliquoted into five different 2 mL eppendorf tubes (Eppendorf Instrumente GmbH, Hamburg, Germany), at concentrations 3000, 1500, 750, 375 or 187 cells/μL. As negative controls, unlabeled cells were introduced into five additional 2 mL eppendorf tubes, with the same cell concentrations as above.

The tubes were examined at 21 °C using a 7 T small animal MRI scanner (Bruker Biospin MRI GmbH, Ettlingen, Germany) equipped with a 40 mm quadrature volume coil. A T_2_ weighted turbo spin echo scan was performed (repetition time (TR) 4000 ms, echo time (TE) 48 ms, field of view (FOV) 2.0 cm, matrix size 256 × 256, slice thickness 1 mm, 15 slices, number of acquisitions (NEX) 4, axial sectioning) to verify homogeneity of the agar/cell solution. Then, a spin echo T_1_ mapping sequence was obtained (TR 5000, 3000, 1500, 800, 400 and 200 ms, TE 33 ms, FOV 3.5 cm, matrix size 256 × 256, slice thickness 1 mm, 1 slice NEX 1). Within the image of each tube, a region of interest (ROI) covering 75% of the surface area of the tube was defined in the scan software (ParaVision 5.1), and T_1_ relaxation values of the tubes were calculated.

### 3.11. Animal Model and Tumor Cell Injections

Twelve-week-old female NOD/SCID mice were bred and maintained in animal facilities under standard conditions, being fed a standard pellet diet and provided water *ad libitum*. The National Animal Research Authority approved all animal procedures (Application No. 20135046, approved 7 May 2013).

H1_DL2 cells were cultured in 175 cm^2^ flasks (Nunc), trypsinated and centrifuged at 900 rpm for four min. The medium was removed, and the cell pellet was dissolved in PBS (Sigma-Aldrich Inc.) at a concentration of 10^7^ cells/mL. The cells were kept on ice prior to injections. Two anaesthetized mice (2% isoflurane) were injected subcutaneously with 0.1 mL cell solution (10^6^ cells) in their left flank or neck using a 30G insulin syringe (Omnican50, B. Brain Melsungen AG, Melsungen, Germany). This is an animal model that is already in use and thus well tested and highly standardized [4,40,44].

### 3.12. Bioluminescence Imaging (BLI) of Tumor Development

After 4 weeks, bioluminescence imaging (BLI) was performed to verify tumor take and growth. This would also suggest that contrast enhancement by MRI could be achieved after administrating the glycogen nanoprobe. Briefly, anaesthetized mice (2% isoflurane) were injected intraperitoneally with 150 mg/kg d-luciferin 10 min prior to whole body imaging, performed with an Optix^®^ MX2 Small Animal Molecular Imager (ART Inc., Saint-Laurent, QC, Canada). Bioluminescent images were acquired with Optix^®^ OptiviewTM (ART Inc.) acquisition software (version 2.00) using an integration time of 0.3 s and a scan resolution of 1.5 mm, and analyzed using Optix^®^ OptiviewTM (version 2.00.01, ART Inc.).

### 3.13. Pilot in Vivo MRI Experiment

*In vivo* MRI experiments were carried out on the mice 8 weeks after tumor cell injection, at which time the tumor sizes were approximately 16 × 9 mm. A 7 T small animal MRI scanner (Bruker Biospin MRI GmbH) equipped with a 72 mm quadrature transmit coil and a mouse brain array receive coil was used. T_2_ weighted spin echo scans were initially acquired with a region of interest over the subcutaneous neck tumors of the mice (TR 4300 ms, TE 36 ms, FOV 2 cm, matrix size 256 × 256, slice thickness 1.5 mm, number of slices 25, NEX 3, axial sectioning). Tumor contrast agent uptake was then studied by T_1_ weighted spin echo scans (TR 1000/740 ms, TE 9 ms, FOV 2 cm, matrix size 256 × 256, slice thickness 1.5 mm, number of slices 11, NEX 6, axial sectioning) before and immediately after administrating 0.5 μmol glycogen nanoprobe or Omniscan (GE Healthcare, Fairfield, CT, USA) through the tail veins. Each animal was first studied with Omniscan, and, after 24 h, the same animal was studied using nanoprobe. T_1_ mapping studies were not performed, to minimize scan time and thus lower the burden to the animals.

The MR images were processed using the ImageJ freeware (version 1.46r, National Institute of Health, Bethesda, MD, USA). Briefly, the regions of interests were carefully outlined around the area containing tumor excluding necrotic tissue and CNR was assessed. CNR in T_1_ weighted MR images obtained after contrast injections were calculated according to the formula: CNR = (ST − SN)/SD, where ST represents mean signal intensity from tumor tissue, SN means signal intensity from normal tissue directly outside the tumor, and SD is the standard deviation of the noise.

### 3.14. Statistical Analysis

Linear regressions and statistical analyses were performed using GraphPad Prism v7 for Mac OS X (GraphPad Software, Inc., La Jolla, CA, USA). Unpaired two-tailed Students *t*-tests were used. Values are presented as mean ± standard deviation (SD). For all tests, a *p* value < 0.05 was considered statistically significant.

## 4. Conclusions

In summary, we have robust data indicating that the nontoxic glycogen nanoprobe is a highly functional imaging probe, both for fluorescence microscopy and MRI. Our data show that the nanoprobe is taken up into the cytoplasm of the tumor cells, and that the probe is likely degraded inside the lysosomes. We now have an excellent foundation upon which we can base further experiments in studies on combined therapeutic and diagnostic issues in metastatic melanoma, including brain metastasis.

## Supplementary Materials

Supplementary materials can be found at http://www.mdpi.com/1422-0067/16/09/21658/s1.

## References

[1] Spano D., Zollo M. (2012). Tumor microenvironment: A main actor in the metastasis process. Clin. Exp. Metastasis.

[2] Fink K.R., Fink J.R. (2013). Imaging of brain metastases. Surg. Neurol. Int..

[3] Giovannini E., Lazzeri P., Milano A., Gaeta M.C., Ciarmiello A. (2015). Clinical applications of choline PET/CT in brain tumors. Curr. Pharm. Des..

[4] Thorsen F., Fite B., Mahakian L.M., Seo J.W., Qin S., Harrison V., Johnson S., Ingham E., Caskey C., Sundstrøm T. (2013). Multimodal imaging enables early detection and characterization of changes in tumor permeability of brain metastases. J. Control. Release.

[5] Jang E.S., Lee S.Y., Cha E.J., Sun I.C., Kwon I.C., Kim D., Kim Y.I., Kim K., Ahn C.H. (2014). Fluorescent dye labeled iron oxide/silica core/shell nanoparticle as a multimodal imaging probe. Pharm. Res..

[6] Xing Y., Zhao J., Conti P.S., Chen K. (2014). Radiolabeled nanoparticles for multimodality tumor imaging. Theranostics.

[7] Khemtong C., Kessinger C.W., Gao J. (2009). Polymeric nanomedicine for cancer MR imaging and drug delivery. Chem. Commun..

[8] Lee D.E., Koo H., Sun I.C., Ryu J.H., Kim K., Kwon I.C. (2012). Multifunctional nanoparticles for multimodal imaging and theragnosis. Chem. Soc. Rev..

[9] Sumer B., Gao J. (2008). Theranostic nanomedicine for cancer. Nanomedicine.

[10] Li N., Yang H., Pan W., Diao W., Tang B. (2014). A tumour mRNA-triggered nanocarrier for multimodal cancer cell imaging and therapy. Chem. Commun..

[11] Laurent S., Saei A.A., Behzadi S., Panahifar A., Mahmoudi M. (2014). Superparamagnetic iron oxide nanoparticles for delivery of therapeutic agents: Opportunities and challenges. Exp. Opin. Drug Deliv..

[12] Jain K.K. (2012). Nanobiotechnology-based strategies for crossing the blood-brain barrier. Nanomedicine.

[13] Horowitz P.M., Chiocca E.A. (2013). Nanotechnology-based strategies for the diagnosis and treatment of intracranial neoplasms. World Neurosurg..

[14] Wang T., Kievit F.M., Veiseh O., Arami H., Stephen Z.R., Fang C., Liu Y., Ellenbogen R.G., Zhang M. (2013). Targeted cell uptake of a noninternalizing antibody through conjugation to iron oxide nanoparticles in primary central nervous system lymphoma. World Neurosurg..

[15] Yuk S.H., Oh K.S., Cho S.H., Lee B.S., Kim S.Y., Kwak B.K., Kim K., Kwon I.C. (2011). Glycol chitosan/heparin immobilized iron oxide nanoparticles with a tumor-targeting characteristic for magnetic resonance imaging. Biomacromolecules.

[16] Chung Y.I., Kim J.C., Kim Y.H., Tae G., Lee S.Y., Kim K., Kwon I.C. (2010). The effect of surface functionalization of PLGA nanoparticles by heparin- or chitosan-conjugated pluronic on tumor targeting. J. Control. Release.

[17] Mansa R., Detellier C. (2013). Preparation and characterization of guar-montmorillonite nanocomposites. Materials.

[18] Wanga C., Huang Y. (2014). Facile preparation of fluorescent Ag-clusters–chitosan-hybrid nanocomposites for bio-applications. New J. Chem..

[19] Win K.Y., Feng S.S. (2005). Effects of particle size and surface coating on cellular uptake of polymeric nanoparticles for oral delivery of anticancer drugs. Biomaterials.

[20] Sakhtianchi R., Minchin R.F., Lee K.B., Alkilany A.M., Serpooshan V., Mahmoudi M. (2013). Exocytosis of nanoparticles from cells: Role in cellular retention and toxicity. Adv. Colloid Interface Sci..

[21] Ridley A.J., Schwartz M.A., Burridge K., Firtel R.A., Ginsberg M.H., Borisy G., Parsons J.T., Horwitz A.R. (2003). Cell migration: Integrating signals from front to back. Science.

[22] Filippov S.K., Sedlacek O., Bogomolova A., Vetrik M., Jirak D., Kovar J., Kucka J., Bals S., Turner S., Stepanek P. (2012). Glycogen as a biodegradable construction nanomaterial for *in vivo* use. Macromol. Biosci..

[23] Naahidi S., Jafari M., Edalat F., Raymond K., Khademhosseini A., Chen P. (2013). Biocompatibility of engineered nanoparticles for drug delivery. J. Control. Release.

[24] Zhang S., Li J., Lykotrafitis G., Bao G., Suresh S. (2009). Size-dependent endocytosis of nanoparticles. Adv. Mater..

[25] Barua S., Rege K. (2009). Cancer-cell-phenotype-dependent differential intracellular trafficking of unconjugated quantum dots. Small.

[26] Kelf T.A., Sreenivasan V.K., Sun J., Kim E.J., Goldys E.M., Zvyagin A.V. (2010). Non-specific cellular uptake of surface-functionalized quantum dots. Nanotechnology.

[27] Chithrani Devika B., Chan W.C.W. (2007). Elucidating the mechanism of cellular uptake and removal of protein-coated gold nanoparticles of different sizes and shapes. Nano Lett..

[28] Rima W., Sancey L., Aloy M.T., Armandy E., Alcantara G.B., Epicier T., Malchere A., Joly-Pottuz L., Mowat P., Lux F. (2013). Internalization pathways into cancer cells of gadolinium-based radiosensitizing nanoparticles. Biomaterials.

[29] Liu J., Bauer H., Callahan J., Kopečková P., Pan H., Kopeček J. (2010). Endocytic uptake of a large array of HPMA copolymers: Elucidation into the dependence on the physicochemical characteristics. J. Control. Release.

[30] Ferrer J.C., Favre C., Gomis R.R., Fernández-Novell J.M., García-Rocha M., de la Iglesia N., Cid E., Guinovart J.J. (2003). Control of glycogen deposition. FEBS Lett..

[31] Glaumann H., Fredzell J., Jubner A., Ericsson J.L.E. (1979). Uptake and degradation of glycogen by Kupffer cells. Exp. Mol. Pathol..

[32] Rejman J., Oberle V., Zuhorn I.S., Hoekstra D. (2004). Size-dependent internalization of particles via the pathways of clathrin- and caveolae-mediated endocytosis. Biochem. J..

[33] Fenouille N., Tichet M., Dufies M., Pottier A., Mogha A., Soo J.K., Rocchi S., Mallavialle A., Galibert M.D., Khammari A. (2012). The epithelial-mesenchymal transition (EMT) regulatory factor SLUG (SNAI2) is a downstream target of SPARC and AKT in promoting melanoma cell invasion. PLoS ONE.

[34] Eikenes L., Bruland O.S., Brekken C., Davies Cde L. (2004). Collagenase increases the transcapillary pressure gradient and improves the uptake and distribution of monoclonal antibodies in human osteosarcoma xenografts. Cancer Res..

[35] Sundstrøm T., Daphu I., Wendelbo I., Hodneland E., Lundervold A., Immervoll H., Skaftnesmo K.O., Babic M., Jendelova P., Syková E. (2013). Automated tracking of nanoparticle-labeled melanoma cells improves the predictive power of a brain metastasis model. Cancer Res..

[36] Terreno E., Geninatti Crich S., Belfiore S., Biancone L., Cabella C., Esposito G., Manazza A.D., Aime S. (2006). Effect of the intracellular localization of a Gd-based imaging probe on the relaxation enhancement of water protons. Magn. Res. Med..

[37] Chacko A.-M., Li C., Pryma D.A., Brem S., Coukos G., Muzykantov V. (2013). Targeted delivery of antibody-based therapeutic and imaging agents to CNS tumors: Crossing the blood-brain barrier divide. Exp. Opin. Drug Deliv..

[38] Morris D.L. (1943). Some effects of the intravenous injection of corn glycogen into rabbits. J. Biol. Chem..

[39] Czerney P. (2011). Dyomics—Colours for life. Fluorescent Dyes for Bioanalytical and Hightech Applications.

[40] Wang J., Daphu I., Pedersen P.H., Miletic H., Hovland R., Mørk S., Bjerkvig R., Tiron C., McCormack E., Micklem D. (2011). A novel brain metastases model developed in immunodeficient rats closely mimics the growth of metastatic brain tumours in patients. Neuropathol. Appl. Neurobiol..

[41] Prasmickaite L., Skrbo N., Høifødt H.K., Suo Z., Engebraten O., Gullestad H.P., Aamdal S., Fodstad Ø., Maelandsmo G.M. (2010). Human malignant melanoma harbours a large fraction of highly clonogenic cells that do not express markers associated with cancer stem cells. Pigment Cell Melanoma Res..

[42] Pawley J.B. (2006). Handbook of Biological Confocal Microscopy.

[43] Kapusta P., Macháň R., Benda A., Hof M. (2012). Fluorescence lifetime correlation spectroscopy (FLCS): Concepts, applications and outlook. Int. J. Mol. Sci..

[44] Daphu I., Sundstrøm T., Horn S., Huszthy P.C., Niclou S.P., Sakariassen P.Ø., Immervoll H., Miletic H., Bjerkvig R., Thorsen F. (2013). *In vivo* animal models for studying brain metastasis: Value and limitations. Clin. Exp. Metastasis.

