# Supplementary material for: A Novel Nanoprobe for Multimodal Imaging Is Effectively Incorporated into Human Melanoma Metastatic Cell Lines
Source: Int J Mol Sci. 2015 Sep 8;16(9):21658–80. doi: 10.3390/ijms160921658 (PMC4613273; doi:10.3390/ijms160921658)
Supplement: Supplementary file 1 [file ijms-16-21658-s001.pdf]

Supplementary Information

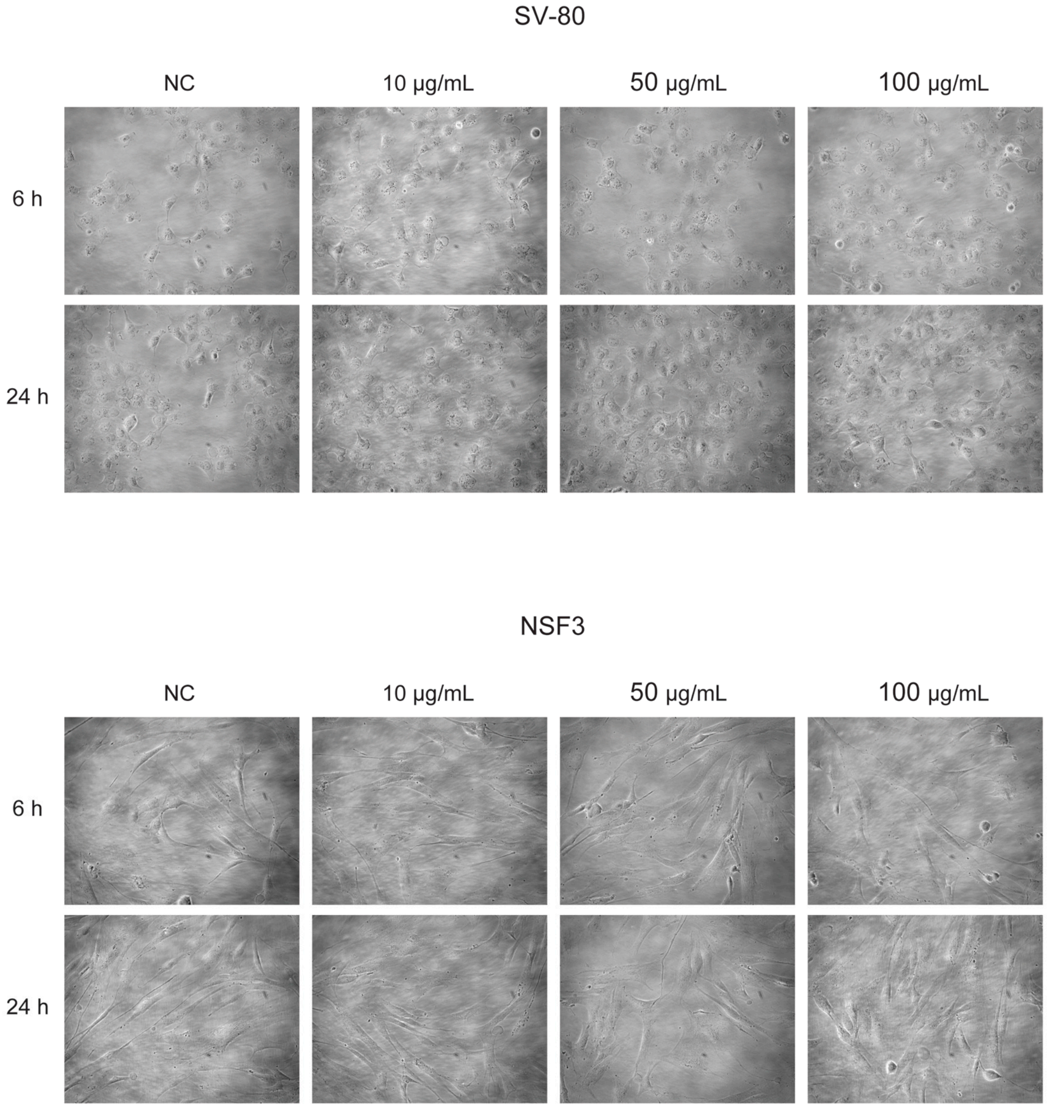

Figure S1. Normal fibroblast cell lines do not internalize the nanoprobe after labeling with up to 100 µg/mL for 24 h. The **upper** panel shows fluorescence images overlaid the light microscopy images for the SV-80 lung fibroblast cell line. No fluorescence can be seen, indicating that the nanoprobe is not taken up by the cells. NC = negative control (no labeling). The **lower** panel shows fluorescence images overlaid the light microscopy images for the NSF3 skin fibroblast cell line. Also here, no fluorescence can be detected, indicating no internalization of the nanoprobe. NC = negative control. For both cell lines, labeling concentrations of 0 (NC), 10, 50 and 100 µg/mL for 6 or 24 h were investigated.
